# Social adaptation in multi-agent model of linguistic categorization is affected by network information flow

**DOI:** 10.1371/journal.pone.0182490

**Published:** 2017-08-15

**Authors:** Julian Zubek, Michał Denkiewicz, Juliusz Barański, Przemysław Wróblewski, Joanna Rączaszek-Leonardi, Dariusz Plewczynski

**Affiliations:** 1 Centre of New Technologies, University of Warsaw, Warsaw, Poland; 2 Faculty of Psychology, University of Warsaw, Warsaw, Poland; 3 Faculty of Mathematics and Information Science, Warsaw University of Technology, Warsaw, Poland; Universite Toulouse 1 Capitole, FRANCE

## Abstract

This paper explores how information flow properties of a network affect the formation of categories shared between individuals, who are communicating through that network. Our work is based on the established multi-agent model of the emergence of linguistic categories grounded in external environment. We study how network information propagation efficiency and the direction of information flow affect categorization by performing simulations with idealized network topologies optimizing certain network centrality measures. We measure dynamic social adaptation when either network topology or environment is subject to change during the experiment, and the system has to adapt to new conditions. We find that both decentralized network topology efficient in information propagation and the presence of central authority (information flow from the center to peripheries) are beneficial for the formation of global agreement between agents. Systems with central authority cope well with network topology change, but are less robust in the case of environment change. These findings help to understand which network properties affect processes of social adaptation. They are important to inform the debate on the advantages and disadvantages of centralized systems.

## Introduction

To study phenomena such as convention sharing, cultural transmission and language evolution researchers are employing multi-agent modeling [1–5]. Such methods allow simulating processes which naturally unfold on very slow time-scales. With computational models it is possible to formulate hypotheses as to how the observed properties of language—such as categories and vocabulary structure—have evolved over time. Realistic models of this kind have to include social factors and limitations, which influence the language structure [6, 7]. But which specific factors are important in this context and when is their influence visible? In our work we contribute to answering this question by studying the effects of social network topology in a multi-agent model of categorization. Through simulations, we investigate the impact of information flow in the network on the quality of the evolved language categories and the stability of the shared communication system in the face of socio-environmental change.

Our starting point is a multi-agent model of categorization developed by Steels and Belpaeme [2]. It strength lies in grounding the evolved categories in external stimuli, providing a link between language and environment. It was originally used to explore the cultural evolution of color categories and vocabulary. The building blocks of this model are simple interaction protocols called *language games* [8]. Language games are designed to represent ecologically important tasks involving language usage. In such game agents interact with each other according to their roles, and afterwards an outcome in the form of communicative success or failure is determined. Each agent adjusts its categories while playing language games in order to maximize its own communicative success. This gradually leads to the emergence of global system of categories shared on the population level. Similarly to the original Steels and Belpaeme’s work, in our simulations agents learn to discriminate and name objects based on their colors. Random color samples from Munsell color chips encoded numerically in CIE LAB space constitute an environment. A system of categories describing the environment is evolved during each simulation.

Network topology determines which agents can interact with each other directly. In the original model a fully connected network was used imposing no restrictions on agents’ communication. This is not a realistic assumption: communication in both artificial and social systems has its costs. It is restricted by geographical, technological, and social factors. We model such restrictions by introducing specific network topologies. Then we ask what properties of such restricted network topologies are desirable for constructing a system of categories useful for communication, which would be robust under perturbations but also flexible, i.e., able to dynamically adapt to varying environmental conditions [7]. In our experiments both the structure of the stimuli (environmental constraints) and the network topology (communication constraints) are prone to change. We address three different questions:

1. How fast, given a certain network topology, the system of categories and vocabulary can achieve global agreement within the whole population?
2. Can a system of categories evolved using a particular communication network topology be adapted when the topology changes so that unrestricted communication between all agents is made possible (fully connected network)?
3. Can an already evolved system of categories be adapted and retrained when the distribution of stimuli occurring in the environment changes? Does the effectiveness of re-training depend on the topology of the network?

The first question is connected to the static situation when the circumstances do not change. The second question is related to change in internal circumstances of the system operation. The third question concerns change in external circumstances. Together they provide a comprehensive account of the issues of social adaptation.

There are existing studies analyzing the impact of network properties, such as size or average node degree, on vocabulary transmission [3, 9, 10]. We are interested in studying the properties related to the concept of network centrality. It can be intuitively understood as the existence of nodes with large influence on the rest of the network (e.g. influential individuals in a social group). Network centrality is an established concept in social studies and has been applied to explain a wide variety of phenomena, such as functioning of an organization [11], decision-making [12, 13], or spread of innovation [14]. Centrality is closely related to network flow [15]. We study two aspects of centrality: *topological centrality*, connected with number of steps needed to propagate information through the network, and *centralization of authority*, connected with the direction of information flow. According to our hypothesis, decentralized network topologies in which information propagation requires smaller number of steps should allow to evolve a consistent set of linguistic categories faster. On the other hand, introducing central authority (forcing information flow from the center toward peripheries) should impose the consistency of categories through the network structure.

To verify our hypotheses we perform experiments with 8 network topologies originally used in social computing experiments by Mason and Watts [16]. All of these networks have uniform node degree 3, but they differ in centrality: each network either maximizes or minimizes certain centrality measure. This results in 4 decentralized networks topologies and 4 centralized topologies. These networks have well-defined theoretical properties and were already studied in social context. To study the effect of the centralization of authority we include star network topology as a special case of centralized network. In this context we analyze different directions of information flow in a network by considering different roles of the central agent in interactions. It can be mostly active (*star speaker*), when it authoritatively forces its own categories on other agents, mostly passive (*star hearer*), when it learns the categories of others, or its role can be balanced (*star balanced*). Using such operationalization we demonstrate that the two aspects of network centrality—topological centrality and centralization of authority—affects the dynamics of categorization system differently. We stress that both aspects of centrality are important factors to be recognized and distinguished.

## Experiment design

The foundation of our research was an agent-based model of categorization utilizing language games developed by Steels and Belpaeme [2] (see Section “Language games model”). During the simulations randomly selected pairs of agents engaged in *guessing game*, in which one of the agents had an active role (*speaker*) and the other a passive role (*hearer*). A simplified version of guessing game in which there was no categorization of stimuli, only naming, was *naming game*. We used this variant as an alternative model to test the universality of our results. Each game could result either in success or a failure (see Section “Language games”). The base measure of agent performance was the fraction of successful guessing games for a given agent over the last 50 games of that agent. Mean value over a population of agents represented their agreement in categorizing stimuli, and was called *communication success* (CS). By analyzing CS it was possible to say how fast the population reaches an agreement.

To answer questions formulated in the introduction we used three experimental designs:

1. To answer question 1) we let the agents learn categories while engaging in interactions according to a specific network topology (optimizing network specific communicative success—CS_S_), but at the same time we measured their communicative success in a fully connected network (global communicative success—CS_G_). Network topologies in which both high CS_S_ and high CS_G_ are achieved were deemed effective in category transmission.
2. Answering question 2) required changing the network topology during the experiment. In the first phase (625 iterations per network node) agents interacted according to a specific network topology, then in the second phase (another 625 iterations per node) they were allowed to retrain their categories with no restrictions on interaction (fully connected network). During both phases CS_S_ on the restricted topology and CS_G_ on the fully connected network were measured. Achieving high CS_G_ after the second phase of the experiment without compromising CS_S_ was a sign that the system of categories developed in the first phase was easily adaptable to unrestricted communication scenario.
3. Question 3) is connected with changing the environment structure while holding the network topology constant. In the first phase (625 iterations per network node) agents learn to categorize stimuli drawn from distribution A, then in the second phase (another 625 iterations per node) they retrain their categories using stimuli drawn from distribution B. Distributions A and B differed significantly (see Section “Environments”). Communicative success is measured using specific network topology and the stimuli from distributions A (CS_A_) and B (CS_B_) separately. Achieving high CS_B_ after the second phase of the experiment without compromising CS_A_ is a sign that the system of categories developed in the first phase was able to adapt to a new environment (adaptiveness) while retaining previous knowledge (retention).

Design 1) is the standard procedure which was applied in the previous simulational studies regarding convention sharing and forming of categories [2, 4, 9]. Designs 2) and 3) allow studying more dynamic properties of the system when the conditions of its operation change. Change of topology may be looked upon as a representation of an internal organizational change, for example when the institution is reorganized and a new hierarchy is introduced. Question whether such transition is easier when the starting point is some specific topology remains an interesting topic in organization science [13].

As for the external environment change a vivid metaphor is a tribe of nomads, which is moving from one climate zone to another. Suddenly they encounter different fauna and flora species and have to coordinate their activity in different set of circumstances than before. To cope with that they have to adapt their language, which involves modifying the vocabulary and the set of categories. The open question is whether network topology affects this process.

### Network properties

In all our experiments a system trained from the beginning using fully connected network topology, as in the original Steels and Belpaeme [2] experiment, was used as a baseline. Since fully connected network provides a direct link between any two nodes, we expected it to produce the best consensus. Our research hypotheses concerned properties of the more specific network topologies.

The first hypothesis was that the efficiency of information propagation in a network is beneficial for building consensus in the three above described scenarios. Possibility to propagate local solutions in a decentralized network should lead to faster convergence. We tested 8 networks either centralized or decentralized according to different centrality measures: betweenness [17], closeness [18], clustering [19], constraint [20].

The second hypothesis concerned the direction of information flow. Basing on the predictions from organizational science [13], we expected that networks in which information is propagated from the center to peripheries (centralized authority) will be more homogeneous and achieve better consensus, but at the expense of some robustness and flexibility. To study these effects we used simple star topology in three different variants:

- *star speaker*—central node has a chance of becoming the speaker in any guessing game it plays proportional to its node degree. For the degree *d* probability of becoming the speaker is $\frac{d-1}{d}$. Here information flows from the center to the peripheries. Central node acts as an authoritative figure enforcing its point of view on the rest of the population.
- *star hearer*—central node has a chance of becoming the hearer in any guessing game it plays proportional to its node degree. For the degree *d* probability of becoming the hearer is $\frac{d-1}{d}$. Here information flows from the peripheries to the center. Central node is a translator mediating between other agents.
- *star balanced*—central node has equal chances of becoming either speaker or hearer. The information flow is balanced.

Regarding the environment, it is known from the previous research that the distribution of stimuli influences the evolved set of categories [21]. We constructed environments A and B in such a way to assure their diversity while not keeping them completely disjoint (see Section “Environments”). It was expected that systems trained on environment A would perform worse on stimuli drawn from environment B, but than can be retrained and adapted to the new environment.

## Materials and methods

### Language games model

As a *stimulus* we understand a vector of numeric values, functioning as a *real-world* reference for agents’ communication (in our case a point in the CIE LAB color space). Stimuli are drawn from a certain distribution called *environment*. Each agent maps any stimuli it receives onto its own internal set of *categories* which initially are arbitrary labels devoid of any intrinsic meaning. Agent adds new categories to its set during the simulation. The particular categories used by different agents are completely independent. An agent maps its categories to a set of *words*—tokens that are shared between agents during a linguistic game.

The simulation consists of a number of iterations. In each iteration two agents interact by playing a *guessing game*. The interaction uses four stimuli sampled from the environment, one of which is arbitrarily marked as the *topic*. One of the agents takes the role of a speaker, who has to communicate to the other—the hearer—which of the stimuli is the topic. It accomplishes this by telling a word it associates with the topic. The hearer then picks the stimulus it thinks is best described by that word (in the given set of stimuli), and if it is the topic, the language game is considered successful. Vocabularies and internal categories of both participating agents are updated depending on the outcome of the game.

Allowing specific network topologies and dynamic changes in topology and environment structure are modifications with respect to the original model [2].

### Agent architecture

**Fig 1:**
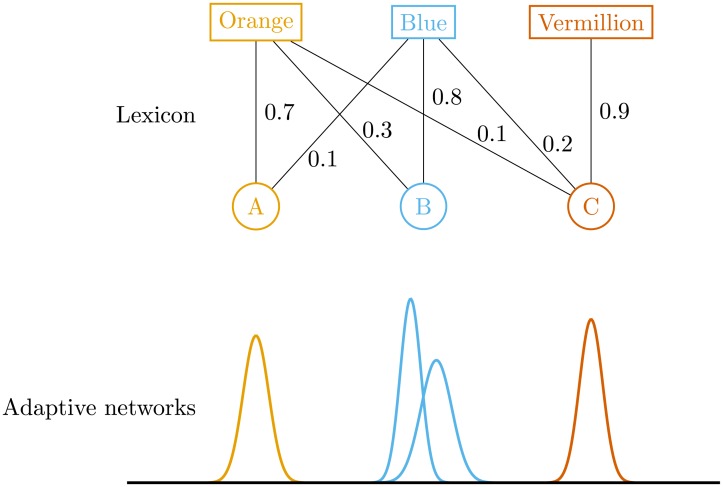
Architecture of an agent.

Architecture of an agent is depicted schematically by Fig 1. Each agent consists of a stimuli categorization system and a lexicon, which maps words to categories. Categorization is implemented using adaptive networks model similar to SUSTAIN categorization model [22]. Each category recognized by an agent is represented by one adaptive network. An adaptive network is composed of multiple reactive units—specialized in detection of a single stimulus and stimuli similar to it. Activation function of each unit is determined by Gaussian function and has the following form:

$$\begin{array}{c} z_{u}\left(x\right)=e^{-\frac{1}{2\sigma^{2}}\sum_{i=1}^{n}{\left(x_{i}-m_{i}\right)}^{2}} \end{array}$$

where **x** is an input stimuli, **m** is a vector of unit’s weights, and *σ*^2^ is the scaling parameter of the kernel (in our experiment *σ* = 1). Reactive units have weights representing their importance for the category. The output of the whole adaptive network for a category *C* has the form:

$$\begin{array}{c} f_{C}\left(x\right)=\sum_{u\in C}w_{u}z_{u}\left(x\right) \end{array}$$

where *w*_*u*_ is the weight of a particular reactive unit. An agent performs classification by assigning a stimulus to the category with the largest reaction scores. In the case of misclassification, agent adds a new reactive unit to the network.

An agent uses associative memory network (lexicon) to relate its internal categories to known words. Each category can be associated with multiple words and vice versa, each association having certain strength. When agent is looking for a category associated with a given word, it chooses a category which has the strongest association with that word. Analogously, while choosing a word for a given category, the one with the strongest association with the category is chosen.

### Language games

In each iteration of the simulation a selected pair of agents plays *guessing game*. One agent is the speaker and the other is the hearer, they act according to their roles. Agents’ adaptive networks and lexicons are modified during the game depending on their interaction with each other and with the environment. A distinguished component of guessing game is a special solo game called *discrimination game*. A simplified variant of the game played by two agents which does not contain discrimination game is called *naming game* (also: *ungrounded naming game*). This game uses only the lexical layer without the categorization layer. All specific parameters of language games, such as the number of stimuli etc., followed the original experiment of Steels and Belpaeme [2].

#### Discrimination game

1. Agent is presented a set of four stimuli with one distinguished stimuli—a topic. Euclidean distance between all possible pairs of stimuli is guaranteed to exceed the value 50.
2. If agent assigns the topic to a different category than the rest of the stimuli game result is SUCCESS.
3. Otherwise game result is FAILURE (see below for consequences of the success and failure of the discrimination game).

#### Guessing game

1. Stimuli are drawn and speaker plays discrimination game. *C*_*s*_ is the category to which the topic was classified. *v* is the word with the strongest association with *C*_*s*_. If there is no word associated with *C*_*s*_, a new word is created.
2. Hearer chooses category *C*_*h*_ with the strongest association with *v* and points to the stimulus which matches *C*_*h*_ the best.
3. If speaker is able to name the topic (using the word *v*) and hearer points to it correctly:
  - Game result is SUCCESS.
  - Speaker increases association strength between *C*_*s*_ and *v* by 0.1.
  - Speaker decreases association strengths between *v* and categories other than *C*_*s*_ by 0.1.
  - Hearer increases association strength between *C*_*h*_ and *v* by 0.1.
  - Hearer decreases association strength between *C*_*h*_ and words other than *v* by 0.1.
  - Adaptive networks of both agents are modified: weights of all reactive units in topic’s category are increased by *βz*_*u*_(**x**_*t*_). **x**_*t*_ is topic, *β* is learning rate (in our experiments *β* = 1).
4. Else:
  - Game result is FAILURE.
  - If speaker cannot discriminate between the topic and other stimuli then:
    - Speaker modifies his adaptive network. If its communicative success is larger than 0.95, the topic is added as a new reactive unit to the category to which it was classified. Otherwise, a new category with a single reactive unit centered at the topic is created.
    - Weights *w*_*u*_ of all reactive units in speaker’s adaptive networks are decreased (forgetting).
  - If hearer does not know the word *v* then:
    - Hearer plays discrimination game, and in case of failure modifies its adaptive network. If its communicative success is larger than 0.95, the topic is added as a new reactive unit to the category to which it was classified. Otherwise, a new category with a single reactive unit centered at the topic is created.
    - Topic’s category from the discrimination game is associated with word *v* with the initial strength 0.5.
    - Weights *w*_*u*_ of all reactive units in hearer’s adaptive networks are decreased (forgetting).
  - If hearer does not point out the topic correctly then:
    - Speaker decreases strength of association between *v* and *C*_*s*_ by 0.1.
    - Hearer decreases strength of association between *v* and *C*_*h*_ by 0.1.
    - Hearer modifies its adaptive network. If its communicative success is larger than 0.95, the topic is added as a new reactive unit to the category to which it was classified. Otherwise, a new category with a single reactive unit centered at the topic is created.
    - Weights *w*_*u*_ of all reactive units in hearer’s adaptive networks are decreased (forgetting).

#### Naming game

1. Stimuli are drawn and speaker chooses a word *v* which has the strongest association with topic (if there is no word associated with topic, a new word is created).
2. Hearer points to the stimulus with the strongest association with *v*.
3. If hearer points to the topic correctly:
  - Game result is SUCCESS.
  - Speaker increases association strength between the topic and *v* by 0.1.
  - Speaker decreases association strengths between *v* and stimuli other than the topic 0.1.
  - Hearer increases association strength between the topic and *v* by 0.1.
  - Hearer decreases association strength between the topic and words other than *v* by 0.1.
4. Else:
  - Game result is FAILURE.
  - If hearer does not know the word *v* then:
    - Hearer associates the topic with word *v* with the initial strength 0.5.
  - If hearer does not point out the topic correctly then:
    - Speaker decreases strength of association between *v* and the topic by 0.1.
    - Hearer decreases strength of association between *v* and the topic by 0.1.

### Social network and information flow

To analyze the role of network centrality in the evolution of categories we used 8 network topologies with 16 nodes of degree 3 embodying different centrality characteristics, as described by Mason and Watts [16]. Based on their centrality four of them are called “centralized” and four “decentralized”. Following the procedure used by the authors we also constructed *max avg bet* and *min avg bet* networks of other sizes (8, 12, 24, 32, 48) to ensure generalization of results.

Role of the direction of network flow was analyzed using simplistic star topology (1 central node connected to *n* neighbors) in three variants: *star speaker* (higher chance of central node acting as speaker), *star hearer* (higher chance of central node acting as hearer), *star balanced* (central node has equal chances of becoming speaker and hearer). We constructed such networks for *n* = 8, 12, 16, 24, 32, 48.

In all experiments a fully connected network was used as a baseline. Various graph properties of these networks are given by Table 1. Network topologies for *n* = 16 are presented visually as Figs A–C in S1 Appendix.

**Table 1:** Structural properties of network topologies used in experiments. The columns: Closeness, Betweenness and Clustering represent average values of respective measure across all nodes.

Network size	Topology	Radius	Diameter	Closeness	Betweenness	Clustering
8	Fully connected	1	1	1.00	0.00	1.00
	Star	1	2	0.59	0.11	0.00
	Max avg betweenness	3	3	0.56	0.13	0.50
	Min avg betweenness	2	2	0.64	0.10	0.12
12	Fully connected	1	1	1.00	0.00	1.00
	Star	1	2	0.56	0.08	0.00
	Max avg betweenness	3	6	0.37	0.18	0.42
	Min avg betweenness	3	3	0.52	0.09	0.00
16	Fully connected	1	1	1.00	0.00	1.00
	Star	1	2	0.55	0.06	0.00
	Max avg betweenness	5	9	0.27	0.20	0.44
	Min avg betweenness	3	3	0.45	0.09	0.00
	Max max closeness	3	5	0.41	0.10	0.06
	Min avg clustering	3	4	0.44	0.09	0.00
	Max var constraint	3	6	0.39	0.12	0.25
	Max avg clustering	6	6	0.31	0.16	0.50
	Min max closeness	5	9	0.27	0.20	0.37
	Max max betweenness	3	6	0.31	0.17	0.37
24	Fully connected	1	1	1.00	0.00	1.00
	Star	1	2	0.53	0.04	0.00
	Max avg betweenness	8	15	0.17	0.23	0.46
	Min avg betweenness	4	4	0.39	0.07	0.00
32	Fully connected	1	1	1.00	0.00	1.00
	Star	1	2	0.52	0.03	0.00
	Max avg betweenness	11	21	0.13	0.23	0.47
	Min avg betweenness	4	4	0.34	0.06	0.00
48	Fully connected	1	1	1.00	0.00	1.00
	Star	1	2	0.52	0.02	0.00
	Max avg betweenness	16	32	0.09	0.24	0.42
	Min avg betweenness	5	6	0.29	0.05	0.00

### Environments

**Fig 2:**
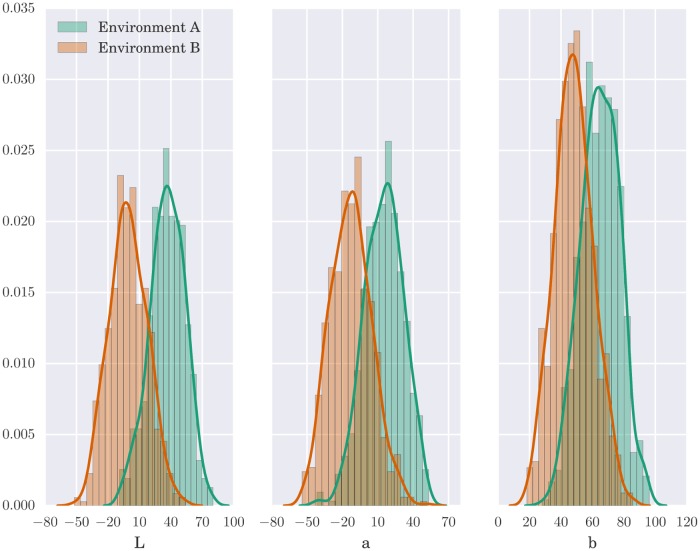
Histograms of L, a, b values for stimuli in environments A and B.

As in Steels and Belpaeme [2] study, in our experiments stimuli were drawn at random from the set of 1269 Munsell color chips. In the experimental condition with environment change we generated two additional sets of 600 chips based on the original one. We modeled the two environments following family resemblance structure with one focal point and stimuli distributed around the focal point according to three-dimensional normal distribution. Such structures are argued to underline many naturally occurring categories [23, 24], and are relatively simple and universal. Environment A was drawn from a distribution centered at point *p*_1_ = (*L* = 66.97, *a* = 18.65, *b* = 38.36), environment B was drawn from a distribution centered at point *p*_2_ = (*L* = 46.24, *a* = −16.46, *b* = −1.41). Variables were uncorrelated, scale in each dimension was 10 times bigger than the difference between *p*_1_ and *p*_2_. This procedure generated two environments containing similar stimuli spanning across whole color space but occurring with different frequencies. Fig 2 presents distributions of L, a, b values in these environments.

For naming game we used simple environment consisting of 16 discrete stimuli. Here also two variants of the environment—A and B—were prepared having 5 stimuli in common. Stimuli were distinguishable for agents but otherwise identical (there was no internal structure of the stimuli).

## Results

### Global agreement of categorization system

The first experiment concerned the ability of different topologies to reach global agreement. We were interested if a categorization system induced by a given topology constituted a good basis for communication between all agents. Since in a single iteration only two agents interact, in order to give larger networks equal chance to propagate information, we decided that the number of iterations should scale with the number of nodes. Hence, all network topologies were trained for 625 simulation iterations per node (5000 for network size 8, 7500 for size 12, 10000 for size 16, 15000 for size 24, 20000 for size 32, 30000 for size 48) using the same set of stimuli. We ensured that after this number of iterations performance of fully connected network converged by comparing obtained CS_G_ scores with those measured at the previous occasion, and not finding statistically significant differences (see Table 2). For each topology and size the simulation was repeated 10 times.

**Table 2:** Results of paired t-test for convergence of CS_G_ for fully connected topology: Scores after 625 iterations per node are compared with scores measured at the previous occasion. There were no significant differences.

Network size	*t*(9)	*p*
8	0.3687	0.7209
12	1.1239	0.2901
16	1.5202	0.1628
24	-0.9338	0.3748
32	-0.7363	0.4803
48	0.1118	0.9134

**Fig 3:**
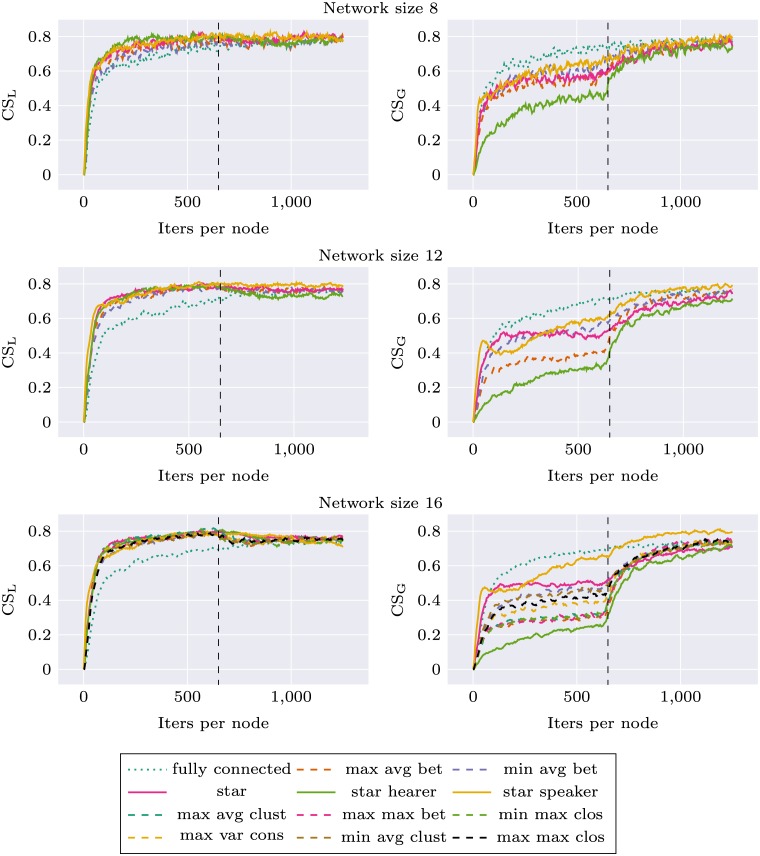
Network specific communicative success (CS_S_) and global communicative success (CS_G_) for different networks, before and after topology change (the change occurs after 625 iterations per node).

**Fig 4:**
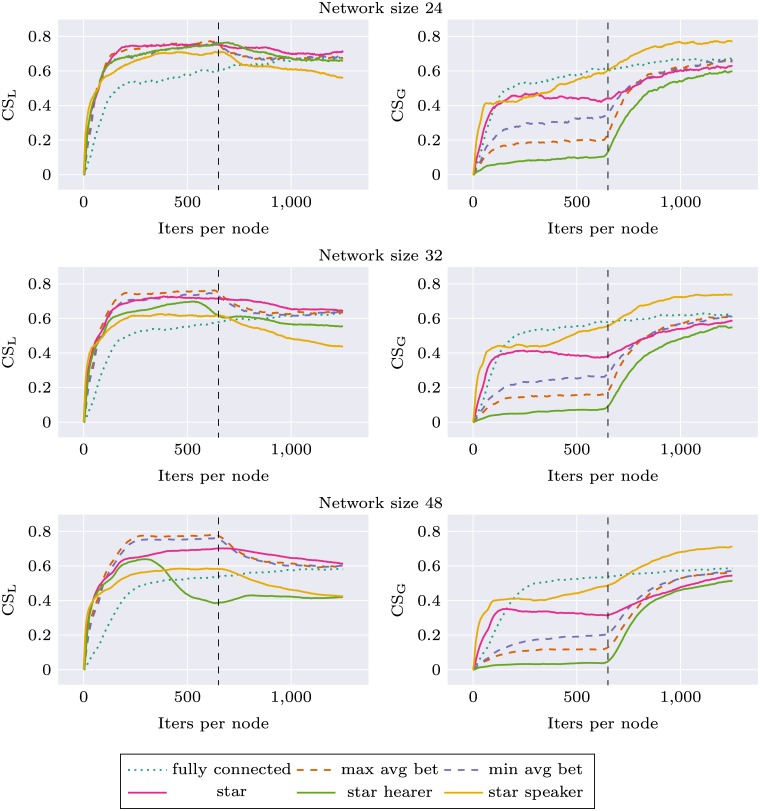
Network specific communicative success (CS_S_) and global communicative success (CS_G_) for different networks, before and after topology change (the change occurs after 625 iterations per node).

Figs 3 and 4 (first half of each plot, before topology change) present the evolution of CS_S_ and CS_G_ during the simulation, for different topologies and network sizes. The general trend is consistent with the expectations: for each topology CS_S_ is higher than CS_G_, CS_G_ is lower than the baseline CS_G_ of the fully connected topology.

For network size 16 we used the original network topologies introduced in Mason’s experiment. We found differences in CS_G_ between decentralized and centralized networks. Topologies *max avg bet*, *max avg clust*, *max max bet*, *min max clos* perform visibly worse than *max var cons*, *min avg bet*, *min avg clust* and *max max clos*. The order of topologies according to CS_G_ is identical to their order according to betweenness centrality measure. The highest scores were obtained for *min avg bet* topology and the lowest for *max avg bet*, both optimizing betweenness. We chose these topologies as the most representative, and in the experiments with other network sizes only these two topologies were used. The results were consistent for all network sizes: the topology with smaller betweenness always outperformed the other one.

To determine statistical significance of these observations, in each simulation we collected CS_G_ scores of various topologies at the point of topology change (625 iterations per node). The results are presented as Fig D in S1 Appendix. Separately for each network size (8, 12, 16, 24, 32, 48) we compared the properties of each topology. In the case of star topology, the three modes of interaction (*star balanced*, *star hearer*, *star speaker*) were treated as different topologies. For each network size we conducted one-way ANOVA comparing the CS_G_ scores of different topologies. Bartlett’s test was used to check the assumption of equal variances between groups. Even though for some network sizes there were significant differences in variances, we decided to proceed normally because of very large differences in means. All ANOVA tests were highly significant (*p* < 0.001, see Table 3). Each ANOVA was followed by a post-hoc analysis, using Tukey’s honest significance test. We used critical values of the test statistic to visualize significant differences between groups [25]. These visualizations are presented as Fig E in S1 Appendix. The previously described differences between topologies were indeed significant.

**Table 3:** ANOVA results for each network size: CS_G_ after 625 iterations per node.

Network size	*F*(5, 54)	ANOVA *p*	Bartlett *p*
8	30.1	< 0.001	0.024
12	108.1	< 0.001	0.084
16	248.3	< 0.001	0.017
24	526.6	< 0.001	0.068
32	940.2	< 0.001	0.067
48	511.7	< 0.001	< 0.001

For network size 16 we also conducted a separate ANOVA, using all 4 centralized and all 4 decentralized networks from Mason’s study. We verified that the variances of scores for different topologies are similar with Bartlett’s test (*χ*^2^ = 8.52, *p* = 0.29, null hypothesis that the variances are equal was not rejected). Then we performed one-way ANOVA test and obtained *F*(7, 72) = 63.95, *p* < 0.001, which was sufficient to state significance difference between topologies. Post-hoc analysis was performed using Tukey’s honest significance test, the results are visualized as Fig F in S1 Appendix.

As can be seen on Figs 3 and 4, there are large differences between the three modes of interaction for the star topology. When the central node performs mostly role of the hearer CS_G_ is very low, while CS_S_ is comparable with the other modes of communication for small networks, and for larger networks (size 32 and 48) it visibly degenerates after some time. This suggests that the central agent is flexible enough to successfully communicate with its peers—without forming global consensus—as long as the number of peers is relatively small. As the number of peers grow, so does the number of categories the central node is required to learn while performing role of the hearer—it is “pulled” simultaneously in different directions which prevents it from forming stable categorization system.

When the central node performs mostly role of the speaker, CS_G_ grows very fast and almost approaches the level of fully connected topology. CS_S_ in smaller networks is similar for all modes of interactions but in larger networks *star speaker* performs worse than balanced *star* which in turn performs worse than *max avg bet* and *min avg bet* topologies. This means that forcing centrally designed categorization on a larger number of agents becomes more difficult, even though it remains a good strategy to build global consensus.

### Adaptation to new network topology

In the second experiment the categorization systems trained with specific network topologies for 625 iterations per node were further retrained for another 625 iterations per node, but using the fully connected topology. Once again, each simulation was repeated 10 times.

A similar analysis as in the previous experiment was conducted—for each network size we compared the CS_G_ of each topology, this time at the end of the second phase, after retraining on the fully connected topology (i.e., after 1250 iterations per node). We used Bartlett’s test to check equality of variances, and then applied ANOVA followed by a pairwise Tukey HSD test. Again, *star balanced*, *star hearer*, *star speaker* were treated as distinct topologies. ANOVA results are provided in Table 4, and the data is visualized as Fig G in S1 Appendix. Results of Tukey HSD test are visualized as Fig H in S1 Appendix.

**Table 4:** ANOVA results for each network size: CS_G_ after topology change (1250 iterations per node).

Network size	*F*(5, 54)	ANOVA *p*	Bartlett *p*
8	1.7	0.143	0.134
12	5.4	< 0.001	0.982
16	8.7	< 0.001	0.934
24	63.2	< 0.001	0.807
32	110.9	< 0.001	0.289
48	103.4	< 0.001	0.748

Figs 3 and 4 present the evolution of CS_G_ and CS_S_ during the simulation. All the systems were flexible enough to adapt to the new circumstances and improve their CS_G_ to be on the same level as the baseline CS_G_ of the fully connected network. This was achieved at the expense of a drop of CS_S_ to the baseline level. There were no apparent differences between different Mason’s topologies, which suggests that centrality of the network used in the first phase does not have an impact on the learning in the second phase.

An interesting effect was observed for *star speaker* topology and larger networks: the performance benefit visible in the first phase propagates to the second phase as well. System which was initially trained using *star speaker* topology has larger CS_G_ in the second phase than the baseline system trained from the beginning using *fully connected* topology. This comes at the expense of CS_S_, which drops below baseline level. Such memory-like effects of increased learning and increased forgetting means that the initially learned structure of categories influences further learning trajectory.

### Adaptation to new environment

**Fig 5:**
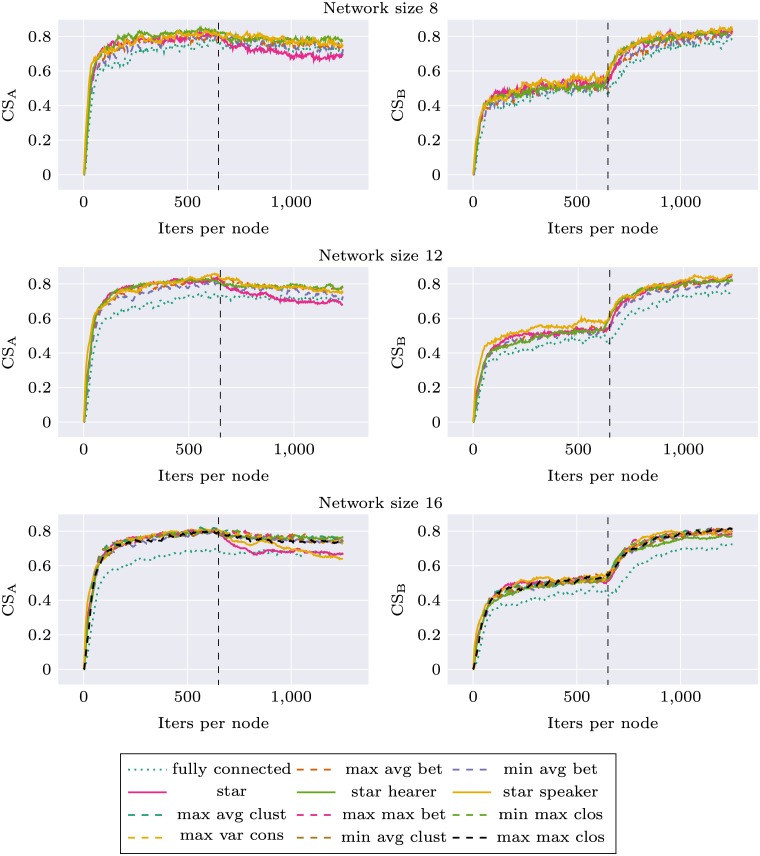
Communicative success (CS) calculated on environment A and environment B for different network topologies. First half of the plot corresponds to a system learning in environment A and the second to the system learning in environment B.

**Fig 6:**
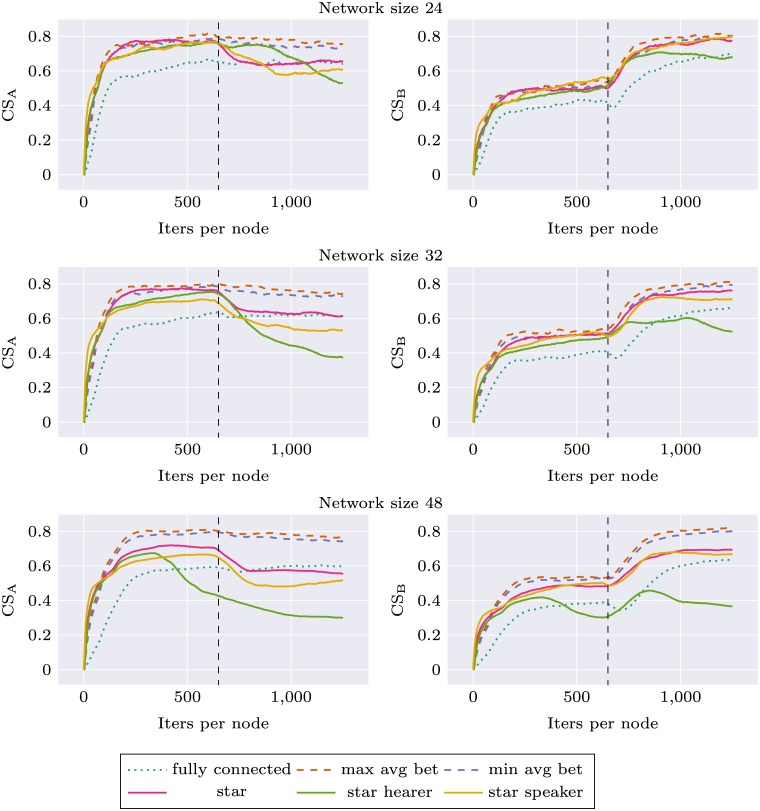
Communicative success (CS) calculated on environment A and environment B for different network topologies. First half of the plot corresponds to a system learning in environment A and the second to the system learning in environment B.

Similar experiments were performed for the environment change condition, where we observed how robust different network topologies are in their adaptation to new stimuli distribution. We used two different environments A and B, as described in Materials section. During the first 625 simulation iterations per node agents were trained on stimuli from environment A, and during the next 625 iterations—on stimuli from environment B. Local communicative success was calculated separately during the whole experiment for two environments (CS_A_ and CS_B_). In this simulation, the network topology was held constant (but various topologies were tested). For each network topology and size, the simulation was repeated 10 times. Results are presented as Figs 5 and 6. For network size 16, performance of all networks with node degree 3 was very similar. We decided to use only *max avg bet* and *min avg bet* topologies in other experiments, and for the sake of visibility only these two topologies are presented on the plots.

Again, we can observe that network specific CS for all topologies is higher than in the case of *fully connected* topology. This applies both to CS_A_ and CS_B_. In the second part of the experiment there is a rapid learning of stimuli from environment B, and the ability to categorize stimuli from environment A deteriorates only very slightly. Interestingly, for *star* topology and larger network sizes this decrease in CS_A_ is much more prominent: *star* performance drops to the baseline level and *star speaker* drops even lower. Performance of *star hearer* again deteriorates for larger networks, in the case of network size 48 even before the shift in environment structure.

This observation was verified through statistical tests as before. We tested differences between CS_A_ for different topologies at the end of the simulation (1250 iterations per node), for each network size separately. The distributions of CS_A_ are shown in Fig I in S1 Appendix. The results are summarized in Table 5. Only in the case of the smallest network (8 nodes) the resulting model was not significant. Pairwise differences in terms of Tukey’s plots are presented as Fig J in S1 Appendix.

**Table 5:** ANOVA results for each network size: CS_A_ after environment change (1250 iterations per node).

Network size	*F*(5, 54)	ANOVA *p*	Bartlett *p*
8	2.8	0.025	0.351
12	11.3	< 0.001	0.797
16	15.8	< 0.001	< 0.001
24	46.0	< 0.001	0.017
32	339.2	< 0.001	0.066
48	327.7	< 0.001	< 0.001

The differences between different topology properties are exacerbated for larger networks. This is to be expected, as the centrality measures exhibit the same behavior, e.g., the betweenness measure for the *min avg bet* and *max avg bet* is 0.13 vs 0.10 for network of size 8, 0.20 vs 0.09 for size 16, and 0.24 vs 0.05 for size 48 (see Table 1). The analysis of those dependencies is beyond the scope of this paper.

These analyses show that categorizations produced with *star balanced* and *star speaker* topologies are unstable, and when the system is retrained with new data—forgetting occurs.

### Categorization layer vs lexical layer

We wanted to verify which of the discovered effects are due to the categorization layer of our model and which can be observed with the lexical layer alone. In other words: are the networks topologies which allow to establish common categories also helpful in establishing common vocabulary in a simplified scenario? Are the obtained results universal or model specific? With these questions in mind we repeated all the experiments using naming game instead of guessing game as the cornerstone of our simulation. The environment was replaced accordingly (see section Materials). We stuck to the rule of running 625 simulation iterations for each network node.

**Fig 7:**
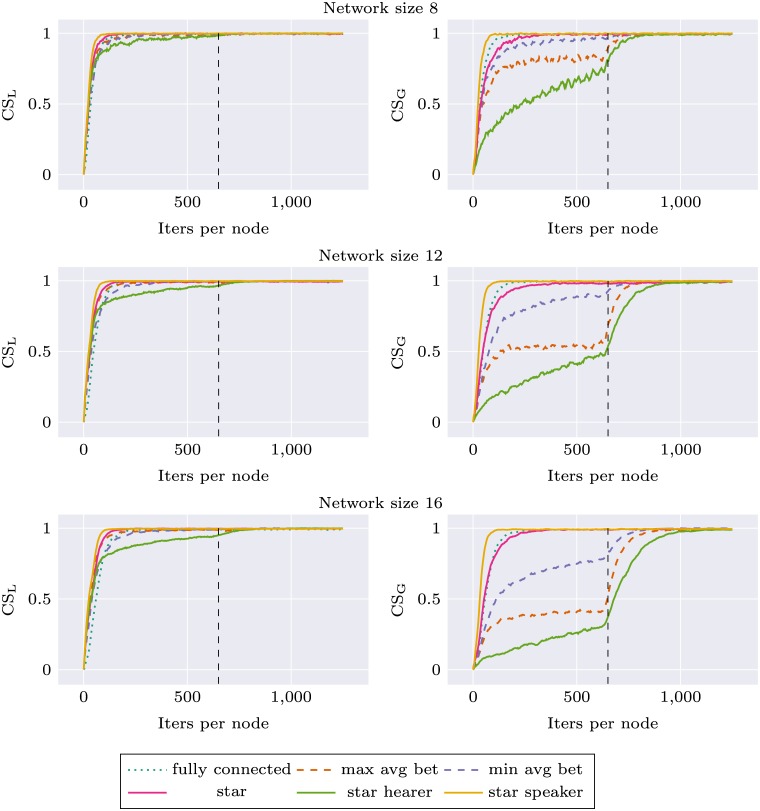
Network specific communicative success (CS_S_) and global communicative success (CS_G_) for different networks before and after topology change, for the naming game only scenario.

**Fig 8:**
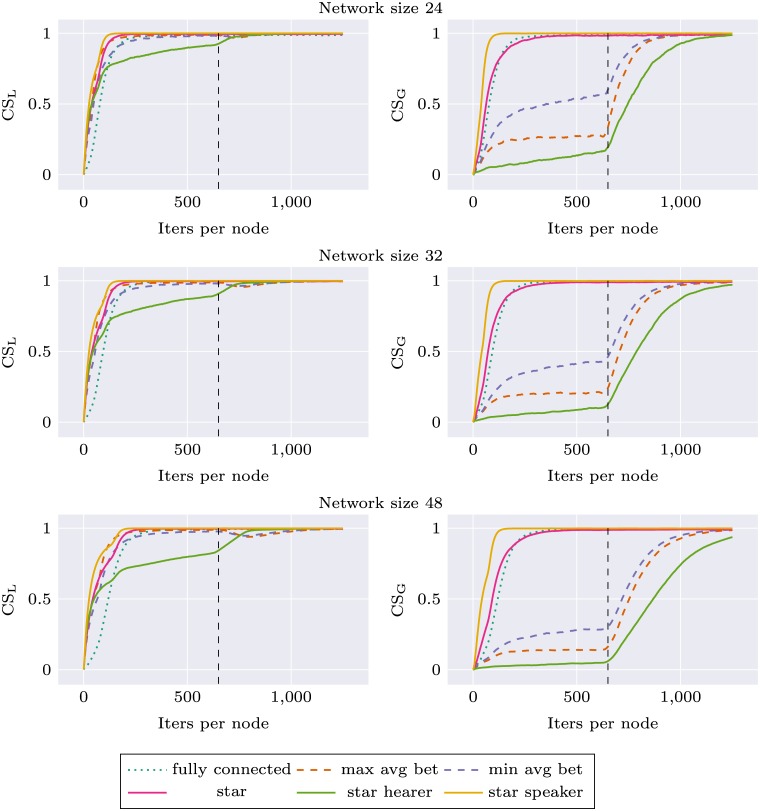
Network specific success (CS_S_) and global communicative success (CS_G_) for different networks before and after topology change, for the naming game only scenario.

Results of the topology shift experiment are presented by Figs 7 and 8. The task is easier than in the case of guessing game and almost all network topologies reach the maximum possible performance (1.0). Perfect CS_G_ score naturally leads to perfect CS_S_ score. Relations between topologies in terms of CS_G_ are preserved: *min avg bet* performs better than *max avg bet*, *star speaker* is quicker to reach maximum performance than *star*, *star hearer* performs the worst. Here *star hearer* is unable to reach maximum performance in terms of CS_S_ score. Very slight forgetting effect is visible for *max avg bet* and *min avg bet* topologies in larger networks. For other topologies this effect is not observed, presumably because of the ceiling effect. Statistical analysis, analogous to those from previous experiments, were conducted for the CS_G_ measure after first phase (625 iterations per node); The results are in the Table 6.

**Table 6:** ANOVA results for each network size: CS_G_ after environment change (1250 iterations per node), for the naming game only scenario.

Network size	*F*(5, 54)	ANOVA *p*	Bartlett *p*
8	124.7	< 0.001	< 0.001
12	606.8	< 0.001	< 0.001
16	2179.0	< 0.001	< 0.001
24	5033.9	< 0.001	< 0.001
32	10403.6	< 0.001	< 0.001
48	35335.4	< 0.001	< 0.001

**Fig 9:**
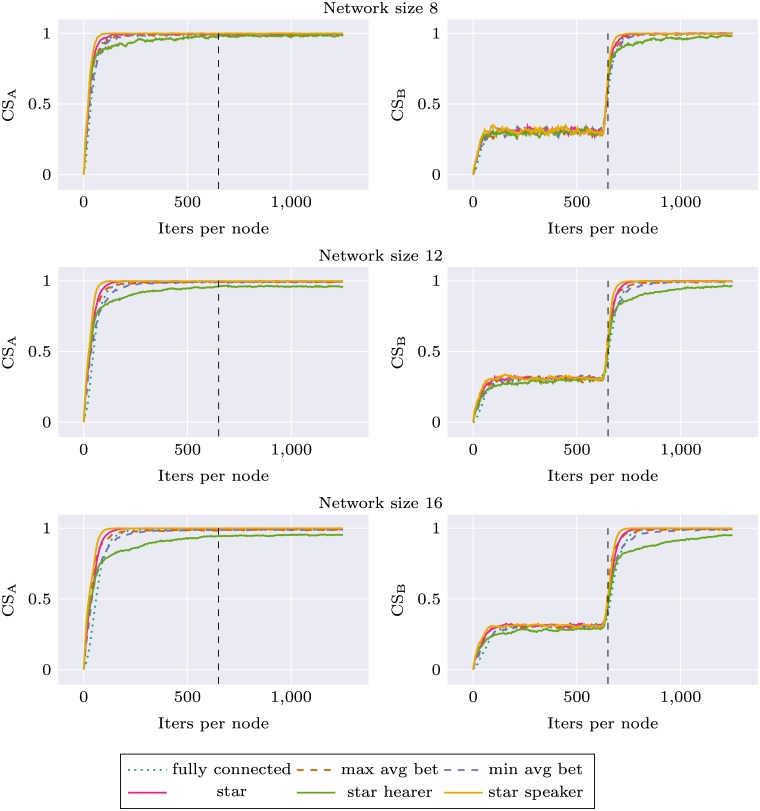
Communicative success (CS) calculated on environment A and environment B for different network topologies, for the naming game only scenario.

**Fig 10:**
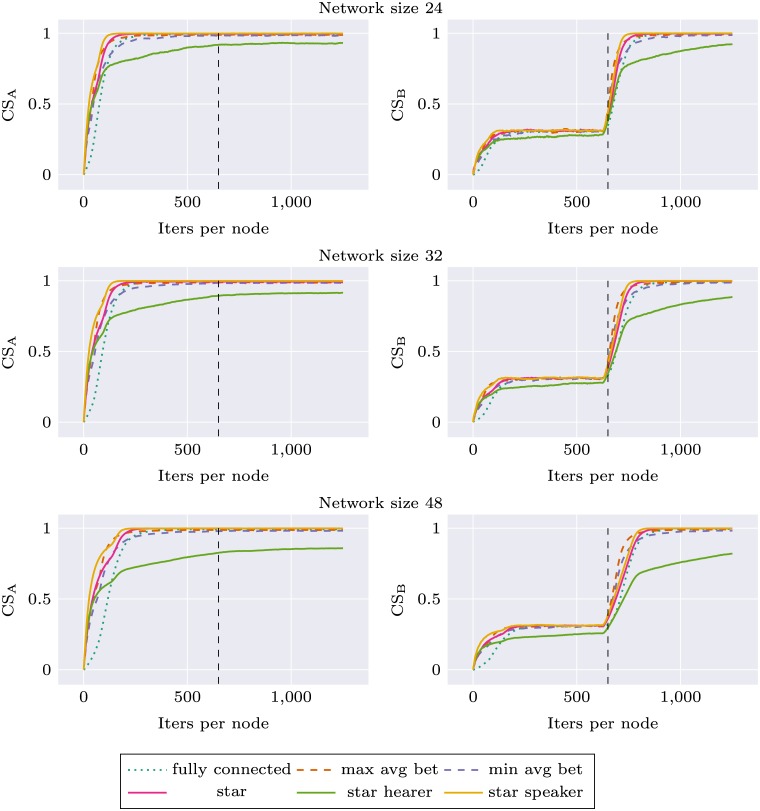
Communicative success (CS) calculated on environment A and environment B for different network topologies, for the naming game only scenario.

Results of the environment shift experiment are presented by Figs 9 and 10. Here the adaptation is very similar for all network topologies except *star hearer* topology, which does not reach maximum performance. The ANOVA results are presented in Table 7.

**Table 7:** ANOVA results for each network size: CS_A_ after environment change (1250 iterations per node), for the naming game only scenario.

Network size	*F*(5, 54)	ANOVA *p*	Bartlett *p*
8	2.8	0.025	0.351
12	11.3	< 0.001	0.797
16	15.8	< 0.001	< 0.001
24	46.0	< 0.001	0.017
32	339.2	< 0.001	0.066
48	327.7	< 0.001	< 0.001

To sum up, effects concerning speed of information propagation (building global consensus) in relation to network centrality and the direction of information flow were visible in the limited experimental situation where only the lexical layer was used. Memory-like effects connected with adaptation and forgetting were visible only when the full model with categorization layer was used.

## Discussion

The results obtained in the above experiments allow for a discussion of the effects of various properties of network topologies manifested in different conditions. Regarding the question concerning transmission of categories through the network we can conclude that it is indeed related to network centrality. High communication efficiency of network topology, indicated by centrality measures, leads to a better global agreement. This is visible as a gap between decentralized and centralized networks global communicative success. Among the centrality measures betweenness was the most informative with regard to global communicative success. It is a promising measure to include in further studies.

Apart from network centrality we also analyzed the direction of information flow in a network. In *star speaker*, information flows from the central node towards the peripheries. This reflects the situation of a central authority imposing words on other nodes, leading to their fast transmission, which is indicated by large global agreement. *Star hearer* has lower global agreement, indicating (relative) lack of common vocabulary, while the symmetric case of *star balanced* topology has intermediate properties. Interestingly however, in terms of network specific success, all three star topologies are similar to each other for small networks, and for larger networks *star speaker* performs worse than *star balanced*. This shows that global unification of symbols (vocabulary) is not necessary for successful communication between local neighbors to occur.

Our findings are consistent with the results of Fagyal et al. [26] who constructed a multi-agent model to investigate linguistic change and the role of central nodes in a network. They confirmed that central nodes were able to influence consensus formation in the network only if they had enough authority, i.e. the information flows from the center to peripheries. This reassures us that the direction information flow is an important factor to include in studies on consensus formation. It may explain some discrepancies in previously published research. In simulations by Richie et al. [10] star topology was characterized by slower convergence of vocabulary than fully connected topology, while Gong et al. [9] reported that agents connected through star topology reached consensus faster than in the case of fully connected topology. We suggest that the difference may be due to different direction of information flow assumed by the authors.

A broader perspective on information flow properties was given by the second part of the experiment, when network topology was changed to a fully connected network. After the topology change, differences in global communicative success disappeared for all but the *star speaker* topology. In this topology, trained agents not only adapted swiftly to fully connected network in the second phase, but also reached performance superior to agents trained with fully connected topology from the beginning. It is worth to stress that global consensus produced by *star speaker* topology in the first phase was already very high, and it probably helped to steer the category formation process in the second phase in the right direction.

The general message of both simulational studies of categorization [2, 9] and experiments with human subjects in social networks [27] is that global consensus can be produced in a distributed manner without any central authority. Our results show that central authority may be nevertheless beneficial for improved coordination. Using authority to build initial skeleton of category system, and then polishing the categories during unrestricted interactions proved to be a successful strategy.

Another properties studied in our experiments were adaptiveness and retention of network performance in the case of environment change. *Star* topologies are characterized by lower retention as compared to uniform-degree topologies—after switching to new environment their performance on the stimuli from the old one deteriorates.

Our results give some general answers regarding the question of desirable properties of network topology in a constructed system. Lack of centralization in topological sense, which helps efficient information propagation, is useful for building consensus. Centralization of authority, understood as information flow from the center to peripheries, may also lead to very good consensus but when the conditions change the dynamics of adaptation of such system will be different. In our experiments *star speaker* topology represented the case of strong central authority, which improved efficiency (high global communicative success) at the cost of smaller retention in the environmental change experiment. We stress that those two notions of centralization are fundamentally different and should not be confused.

This fits into a broader discussion on the merits of centralized vs decentralized systems. On the technical level engineers speak about advantages of decentralized software solutions. It is visible for instance in the context of resource management in distributed computational grids, where centralized systems are perceived as easier to construct but less robust and less flexible [28, 29]. On the higher level there is an old debate on decentralizing information systems [30]. Hugoson [31] claims that centralized systems are often introduced as a way to ensure consistency and order, but as the systems grow they are becoming very hard to manage, and adapt to the changing needs. Decision making processes within an organization can also be subject to centralization. According to Andrews et al. [12] organizations with highly centralized authority focus more on improving the efficiency of their existing operation, while decentralized organizations exhibit greater flexibility in their constant search of new opportunities [12]. Those examples come from different domains with their own characteristics but there is a single common theme: a trade-off between controlling internal dynamics of the system (ensuring consistency and consensus) and maintaining flexibility when facing a change in external conditions. Systems with centralized authority, like *star speaker* topology in our experiments, tend to cope with the internal changes better than with the external ones. The notion of internal vs external change may be useful for analyzing dynamical systems of this kind, and could be elaborated upon in further works.

## Conclusions

We have shown that the language games model can be used with a range of network topologies, and is flexible enough to deal with environment or topology changes during the simulation. Topological network centrality and the direction of information flow were identified as two distinct factors with significant impact on the characteristics of the evolved categorization system.

Fully connected network topology proved to be the most effective in transmission of categories. On the local level however, more restricted topologies achieved high network specific communicative success without forming global agreement. Since in real-world situations the fully connected topology can be prohibitively expensive, this result may be informative for designing similar systems, under the assumption that communication effectiveness, rather than the exact category transmission, is required.

Our results reinforce confidence in the usefulness of the language games model in studying language phenomena, also in more realistic, non-static context. In turn, further research based on multi-agent modeling and comparative paradigm can help to better understand the relationship between properties of a system and circumstances that created it (e.g. network topology and its evolution). These factors can then be summarized as network centrality measures, like betweenness, allowing simple evaluation and comparison. Such experiments are a good way of preliminary hypotheses testing, which informs research of real communicative systems, both natural and artificially designed.

## Supporting information

S1 Appendix Supporting figures. (PDF)
