# Supplementary material for: Social adaptation in multi-agent model of linguistic categorization is affected by network information flow
Source: PLoS One. 2017 Aug 15;12(8):e0182490. doi: 10.1371/journal.pone.0182490 (PMC5557553; doi:10.1371/journal.pone.0182490)
Supplement: S1 Appendix — (PDF) [file pone.0182490.s001.pdf]

Social adaptation in multi-agent model of linguistic categorization is affected by network information flow

S1 Appendix. Supporting figures

Julian Zubek, Michał Denkiewicz,
Juliusz Barański, Przemysław Wróblewski,
Joanna Rączaszek-Leonardi, Dariusz Plewczynski

July 22, 2017

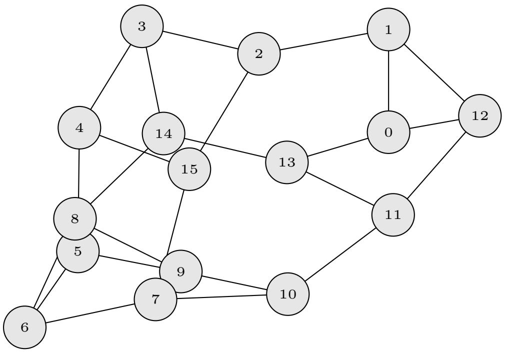

Max max closeness

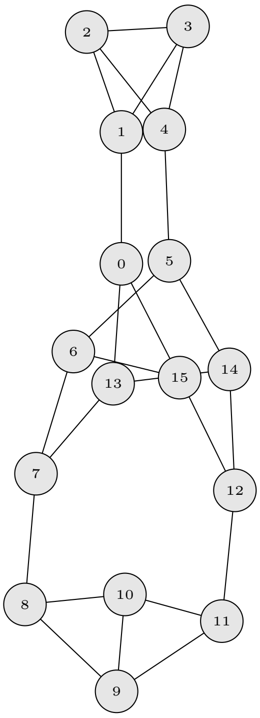

Max var constraint

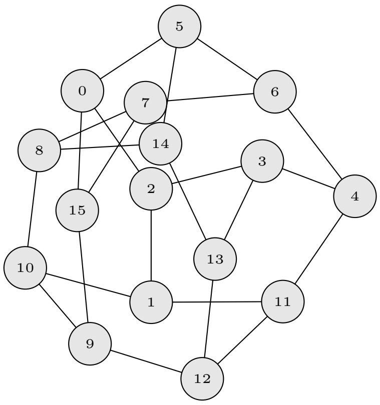

Min avg betweenness

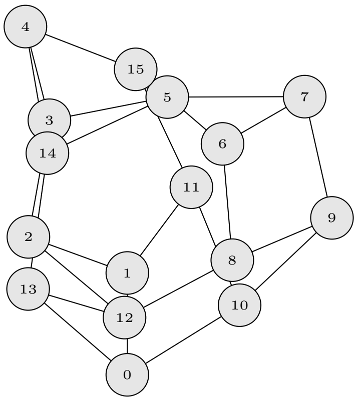

Min avg clustering

Figure A: Decentralized network topologies.

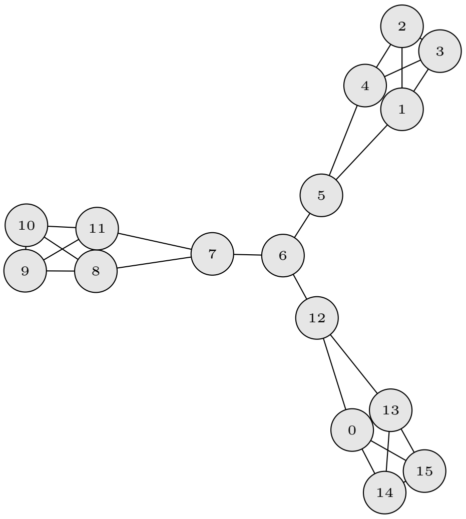

Max max betweenness

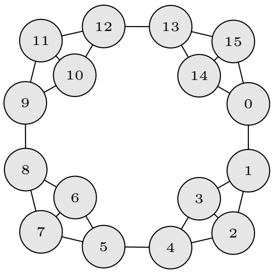

Max avg clustering

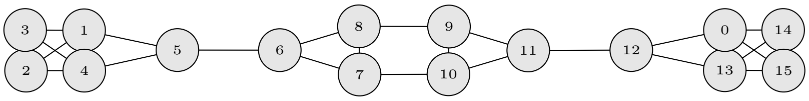

Min max closeness

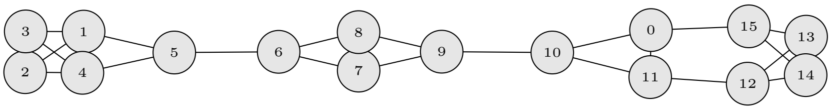

Max avg betweenness

Figure B: Centralized network topologies.

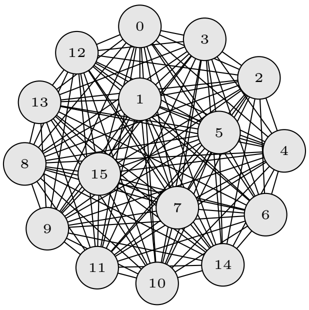

Clique

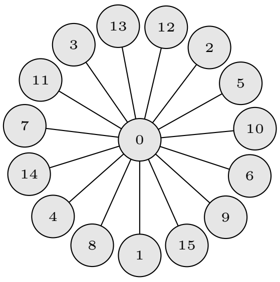

Star

Figure C: Fully connected and star network topologies.

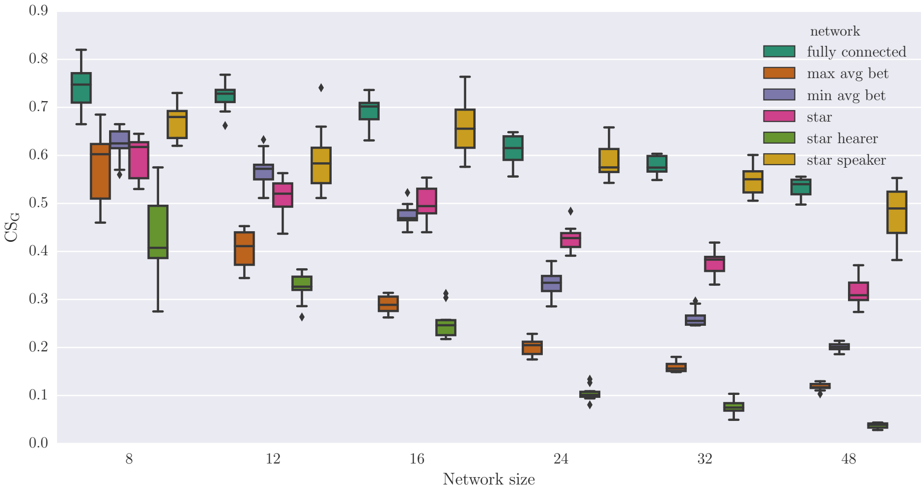

Figure D: Distributions of CS_G scores after 625 iterations per node, for different topologies and network sizes.

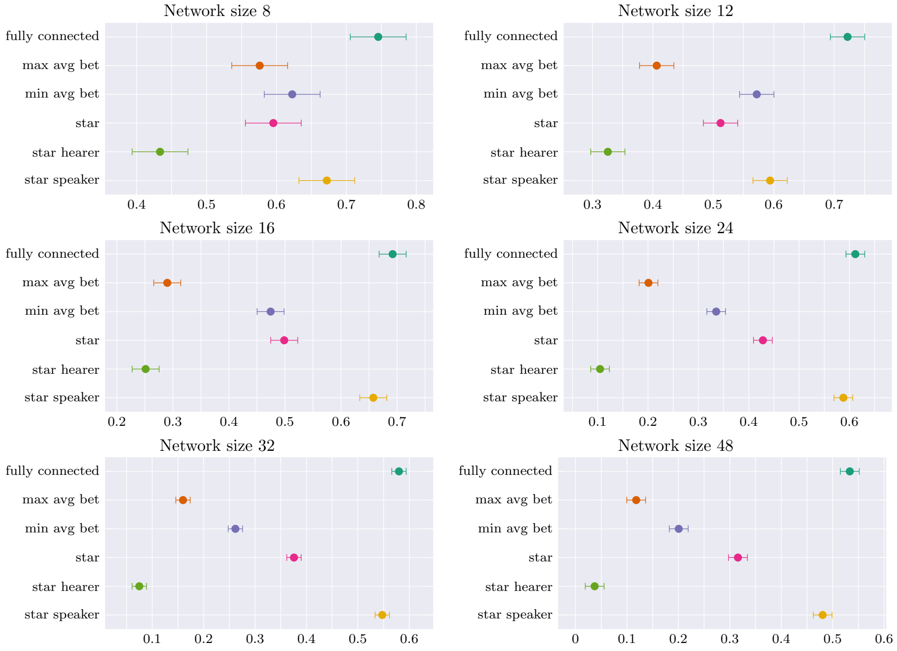

Figure E: Differences between CS_G scores after 625 iterations per node between different topologies tested with Tukey's honest significance test at significance level 0.05. Overlapping intervals for two topologies mean that there is no significant difference between them.

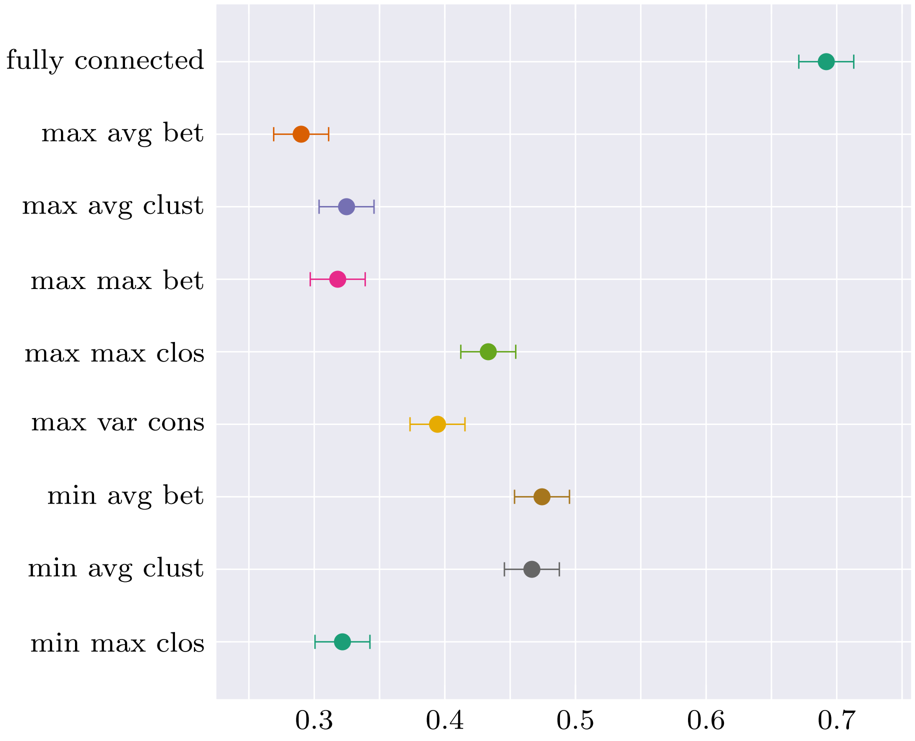

Figure F: Differences between CS_G scores after 625 iterations per node, tested with Tukey's honest significance test at significance level 0.05. Overlapping intervals for two topologies mean that there is no significant difference between them.

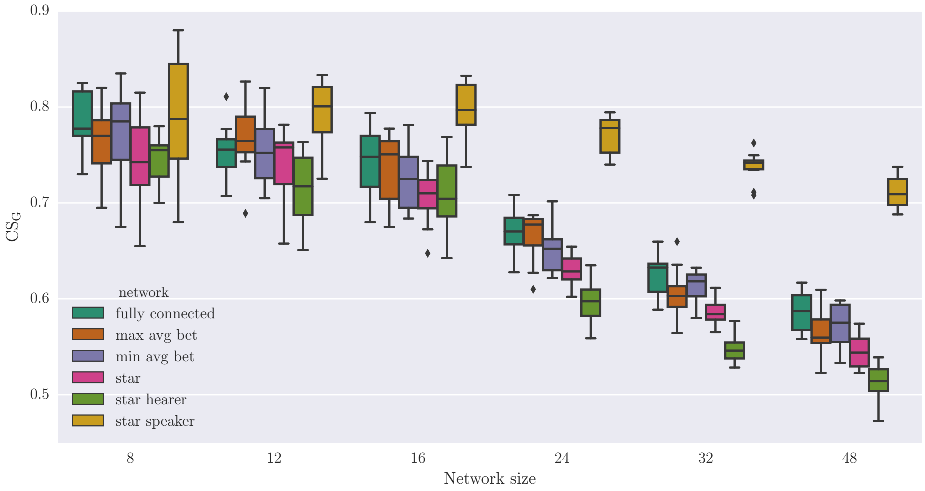

Figure G: Distributions of CS_G scores after 1250 iterations per node for different topologies and network sizes, for the topology change experiment.

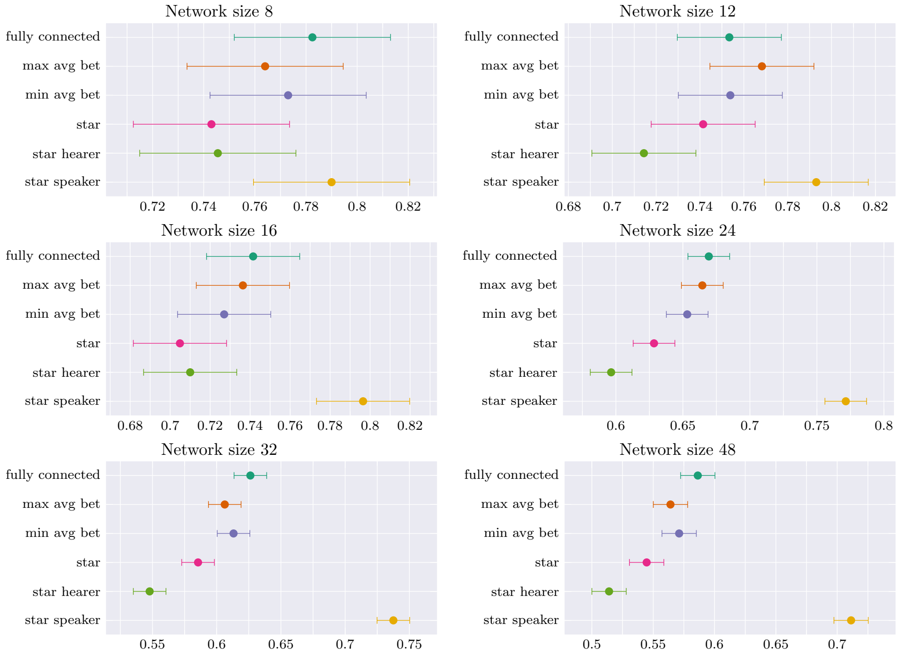

Figure H: Differences between CS_G scores after 1250 iterations per node for the second (topology change) experiment, tested with Tukey's honest significance test at significance level 0.05. Overlapping intervals for two topologies mean that there is no significant difference between them.

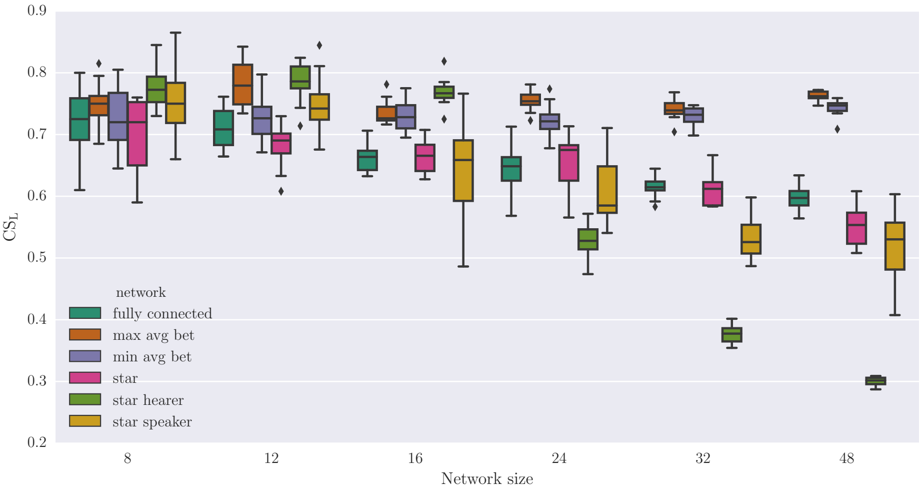

Figure I: Distributions of CS_A scores after 1250 iterations per node for different topologies and network sizes, for the environment change experiment.

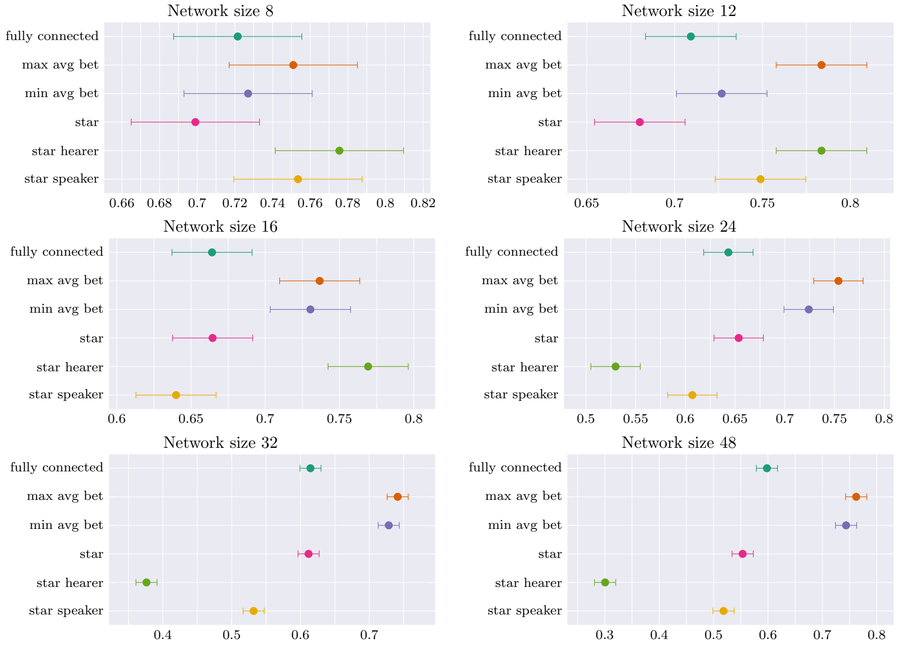

Figure J: Differences between CS_{A} scores after 1250 iterations per node for the third (environment change) experiment, tested with Tukey's honest significance test at significance level 0.05. Overlapping intervals for two topologies mean that there is no significant difference between them.
